# Effect of Danhong injection on neurological recovery and adverse events in patients with acute ischemic stroke: A protocol for a randomized, double-blind, placebo-controlled clinical study

**DOI:** 10.1097/MD.0000000000027683

**Published:** 2021-11-19

**Authors:** Junfeng Gao, Xiangzhong Shao, YiXiang Guan, Juxiang Mei

**Affiliations:** Hai’an Hospital Affiliated to Nantong Medical College, Hai’an, Jiangsu Province, China.

**Keywords:** Danhong injection, ischemic stroke, protocol, randomized controlled trial

## Abstract

**Background:**

Acute ischemic stroke (AIS) is characterized by high disabling and recurrent recurrence, and its severe neurological impairment and vascular adverse events (AEs) limit the recovery of patients. Danhong injection is a complementary alternative to the treatment of AIS, and previous studies have demonstrated its efficacy and safety. However, there is no long-term follow-up and rigorous clinical study to evaluate the effect of Danhong injection on neurological recovery and AEs in patients with AIS.

**Methods:**

This is a prospective randomized, double-blind, placebo-controlled trial investigating the effect of Danhong injection on neurological recovery and AEs in patients with AIS. Participants were randomly divided into treatment and control groups in a 1:1 ratio. The treatment group was treated with Danhong injection and the control group were treated with placebo under the guideline recommended basic treatment. After 14 days of continuous treatment, the follow-up period was 6 months. Observation indicators include: National Institute of Health Stroke Scale, modified Rankin scale, symptomatic intracranial hemorrhage, the incidence of new major vascular events within 6 months, and all-cause mortality. Finally, the data were analyzed statistically using the SPASS 22.0 software.

**Discussion:**

This study will evaluate the effect of Danhong injection on neurological recovery and AEs in AIS. The results will provide a reference for the clinical use of AIS.

## 1 Introduction

Globally, stroke is one of the major causes of death and disability.^[1]^ Ischemic stroke is a common type of stroke, accounting for about 60% to 80% of all strokes,^[2]^ referring to the brain dysfunction caused by ischemia of brain tissue and hypoxia.^[3]^ Ischemic stroke is highly disabling and prone to recurrence, which can seriously damage the patient's central nervous system and even lead to death.^[4–6]^ It is estimated that about 6.2 million deaths worldwide die from ischemic stroke every year,^[7]^ and the number is still rising, putting a heavy burden on families and public health care.^[8]^

The key to acute ischemic stroke (AIS) treatment is the early and effective realization of vascular recommunication and restored brain tissue irrigation,^[9]^ which protects brain tissue and promotes brain metabolism. Intravenous thrombolysis with recombinant tissue plasminogen activator in time window is currently internationally recognized as an effective treatment for AIS,^[10]^ however, since the thrombolysis time window is narrow, and there are bleeding complications,^[11]^ only a few patients can receive thrombolytic therapy. Data from the Chinese National Stroke Registry showed 21.5% of acute stroke patients attended the emergency department within 3 hours, 12.6% were eligible for thrombolytic therapy, but only 1.6% received intravenous recombinant tissue plasminogen activator.^[12]^ Therefore, it is still necessary to continue to explore drugs with wide clinical adaptability and high safety.

In China, traditional Chinese medicine (TCM) for promoting blood circulation and removing blood stasis is widely used in the treatment of ischemic stroke. TCM injections with Danshen (*Radix Salviae Miltiorrhiae*) as the main component are commonly used to treat AIS due to its remarkable efficacy and rapid effect.^[13]^ Danhong injection is a representative drug and has been approved by the China Food and Drug Administration for the treatment of AIS.^[5]^ Danhong injection, composed of Danshen (*Radix Salviae Miltiorrhiae*) and Honghua (*Flos Carthami*), is widely used in the treatment of cardiovascular and cerebrovascular diseases.^[14]^ It has shown that Danhong injection can effectively inhibit platelet aggregation, reduce arterial inflammatory factor levels, up-regulate endothelin-1 levels, regulate blood lipid, inhibit atherosclerosis progression, stabilize atherosclerotic plaque, improve brain microcirculation, and relieve ischemia and reperfusion injury.^[15]^ Experimental studies confirmed that Danhong injection can improve cerebral ischemia reperfusion-injury through its anticoagulation, antithrombosis, antifibrinysis, and antioxidant activity and exerts neuroprotective effects by reducing the Bax Immunoreactivity and increasing Bcl-2 expression.^[16]^ Although the current studies have confirmed the efficacy and safety of Daninjection in the treatment of AIS,^[15]^ a long-term follow-up is lacking. Approximately 10% of patients with AIS die within 1 year, and 20% of ∼ 25% of patients will develop severe disabilities. Due to the lack of long-term follow-up observations, it is still debated whether Danhong injection can reduce the occurrence of adverse events (AEs) and promote neurological recovery in AIS patients. This study will explore the effect of Danhong injection on neurological recovery and AEs in AIS patients through a prospective randomized, double-blind, placebo-controlled trial.

## 2 Materials and methods

### 2.1 Study design

**Figure 1:**
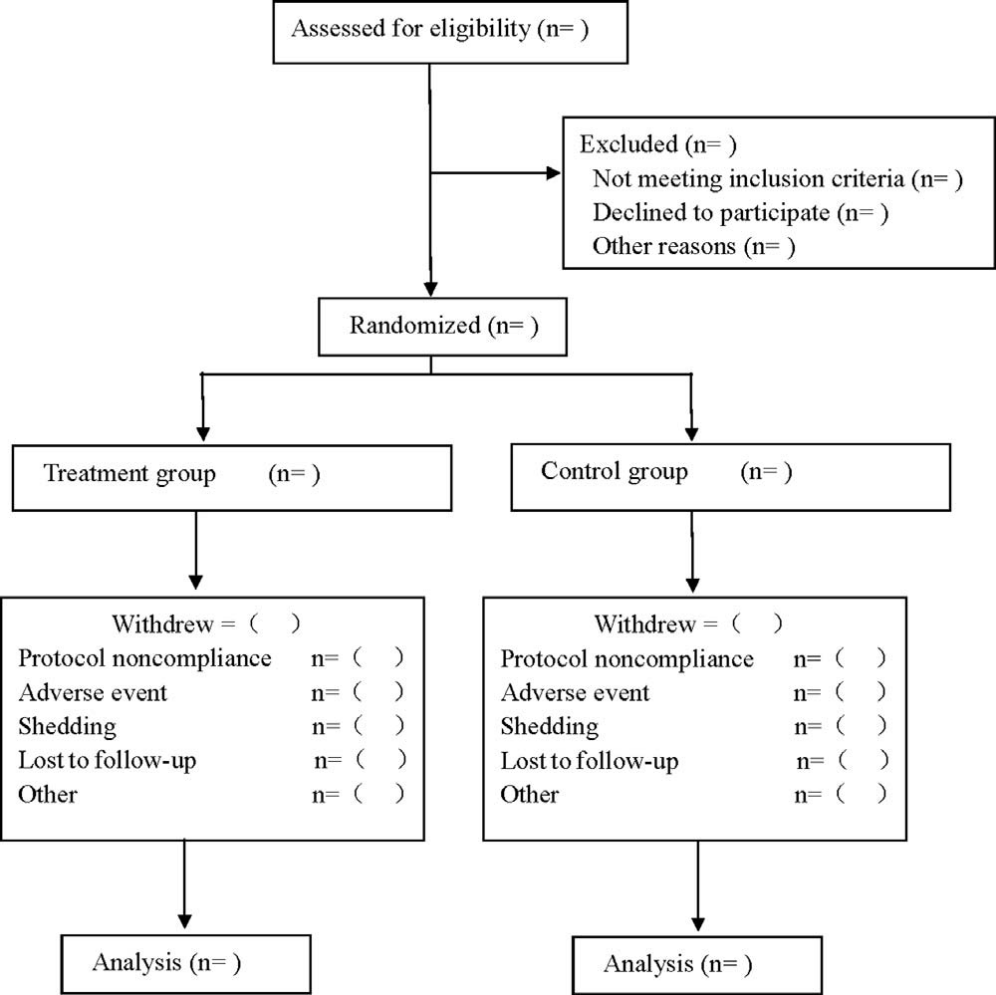
Flow diagram of this clinical trial.

This was a prospective randomized, double-blind, placebo-controlled study investigating the effect of Danhong injection on recovery of neurological function and AEs in AIS patients. Participants were randomly divided into treatment and control groups in a 1:1 ratio. The treatment group was treated with Danhong injection and the control group were treated with placebo. After 14 days of continuous treatment, the follow-up period was 6 months, see Figure 1 for flow diagram and Table 1 for study schedule.

**Table 1:** Study schedule.

	Stage
	Screening period	Treatment period		Follow-up		
Project	Baseline	1-week	2-week	1-month	3-month	6-month
Record fill	√					
Fulfill inclusion criteria and exclusion criteria	√					
Sign informed consent	√					
Random allocation	√					
Treatment	√	√	√			
Effectiveness observation						
NIHSS	√		√	√	√	√
mRS					√	
SICH				√	√	√
New major vascular events				√	√	√
All-cause mortality				√	√	√
Safety evaluation						
Blood test and urinalysis	√		√			√
Liver and kidney function	√		√			√
Record of adverse event		√	√	√	√	√

mRS = modified Rankin scale, NIHSS = National Institute of Health Stroke Scale, SICH = symptomatic intracranial hemorrhage.

### 2.2 Ethics and registration

This study protocol would be performed in accordance with the Declaration of Helsinki and the Ethical Guidelines for Clinical Research. This study protocol would also be in strict accordance with the latest Consolidated Standards of Reporting Trials (2017) and Standard Protocol Items: Recommendations for Interventional Trials 2013 statement specification. This study has been reviewed by the Clinical Research Ethics Committee of our hospital and registered with open science framework (registration number: DOI 10.17605/OSF.IO/79USM). All patients would sign a written informed consent form before the study begins, and they could discontinue or drop out of the trial at any time during the study.

### 2.3 Patients

#### 2.3.1 Diagnostic basis

The diagnosis of AIS was referred to the description of the acute phase of ischemic stroke in the *Guidelines for the Diagnosis and Treatment of Acute Ichaemic Stroke in China* (2014 edition).^[18]^

#### 2.3.2 Inclusion criteria

(1) Age ≥18 years, and ≤70 years;
(2) Meet the diagnostic criteria of AIS, with symptoms within 24 hours, and bleeding was excluded by cranial MRI or CT examination;
(3) National Institute of Health Stroke Scale (NIHSS) score ≥5 and ≤20;
(4) First disease onset;
(5) Subjects agreed to join the study and signed the informed consent form.

#### 2.3.3 Exclusion criteria

(1) CT or MRI examination showed the presence of other brain diseases (such as vascular malformation, tumor, abscess, or multiple sclerosis, etc);
(2) Patients with planning or already receiving thrombolytic or intravascular therapy;
(3) Patients with previous central nervous dysfunction or motor dysfunction due to stroke or other diseases;
(4) ALT, AST or Cr reached 1.5 times the normal upper limit^[19]^;
(5) Patients with other diseases that may increase the risk of bleeding, such as a history of important organ bleeding (e.g., cerebral bleeding, upper gastrointestinal bleeding), platelet count reduction, abnormal coagulation function, recent active bleeding, etc;
(6) Women with pregnancy, preparation for pregnancy, or lactation;
(7) Patients allergic to drug used in the study;
(8) Those who have attended or were participating in other clinical trials in nearly a month.

#### 2.3.4 Criteria for shedding

(1) If AEs or serious adverse event (SAEs) occurred, the researcher should suspend or stop the study according to the condition and give the corresponding treatment to the patient;
(2) The patient's condition has not improved or deteriorated during the study period. Although the prescribed treatment or follow-up has not been completed, in order to protect the subject, the subject should withdraw from the trial and receive other effective treatment;
(3) The study results were affected by the poor compliance of subjects, who changed drugs or added non-prescribed drugs;
(4) For any reason, the subject was unwilling or impossible to continue with the clinical trial and asked the investigator to quit the trial;
(5) The patient was accidentally included after misdiagnosis, or did not meet the inclusion and exclusion criteria.

For midway withdrawal cases or missed cases, the researcher should take active measures to complete the last test as far as possible to analyze their efficacy and safety, and take corresponding treatment measures. All shedding cases should be filled in the case report form (CRF) for the cause of case shedding.

### 2.4 Sample size

The sample size estimate was based on the mean and standard deviation of the patients’ NIHSS^[20]^ scores 14 days after treatment, referring to pre-experimental results, 19.51 ± 6.37 for the treatment group and 15.43 ± 5.62 for the control group were worked out. Set α = 0.025, one-sided test, *β* = 0.10. Forty-seven participants per group were calculated by PASS15.0 software, with an estimated exit rate of 10%, and 53 patients would be included in per group.

### 2.5 Randomization

Patients who met the criteria would be randomized into the treatment group (Danhong injection group) or the control group (placebo group) in a 1:1 ratio. The randomization procedure would generate randomized numbers using SAS 9.3 software (SAS Institute, Cary, NC) via a central network-based randomized tool by independent statisticians not involved in trial implementation or statistical analysis. The research assistant entered patient information on the tablet and was assigned a random number to complete the random assignment according to the random number.

### 2.6 Blinding

Throughout the study, all patients, treating physicians, and statisticians were agnostic about the randomly assigned outcome. Considering that Danhong injection is different in color from 0.9% saline, we sealed the drop bottle using an opaque brown bag and injected it using a brown infusion device. A professional nurse was responsible for preparing the drugs in a special infusion room and sealing the infusion bottle with brown bags with only the number corresponding to the patient. One nurse was responsible for completing the infusion according to the number, and 2 professional nurses who could not be contacted independently performed the above procedures. They were all required to sign a confidentiality agreement before the study begins.

### 2.7 Interventions

Patients in both groups were provided with basic treatment and standard care in strict accordance with the *Chinese Guidelines for the Diagnosis and Treatment of Acute Ischemic Stroke (2018)*.^[21]^ It included basic treatment such as controlling body temperature, blood pressure, and blood sugar, improved brain blood circulation, antiplatelet aggregation, antibrain edema, and nutritional support treatment. All of these conventional treatments were recorded in detail in the CRF. During the study period, medication needed by patients for other underlying diseases was also documented in detail in the CRF. However, any other TCM with similar pharmacological effects as Danhong injection was not allowed throughout the study period.

The treatment group was performed with Danhong injection (Danhong Pharmaceutical Co., Ltd, Shandong, China), 40 mL/d, combined with 0.9% saline, 250 mL/d, intravenous infusion, and control groups with placebo (0.9% saline, 40 mL/d, combined with 0.9% saline, 250 mL/d, i.v. All patients were given standard treatment for 14 days of treatment.

### 2.8 Outcomes

(1) Primary outcomes:
  i. NIHSS,^[20]^ NIHSS is a tool used to objectively quantify neurological impairment after stroke, and the higher the score indicates that the worse the neurological deficit;
  ii. modified Rankin scale (mRS),^[22]^ the mRS score was assessed from patient onset to day 90. mRS is a commonly used scale to measure disability or dependence in daily activities in patients with stroke or other neurological dysfunction. The scale ranges from 0 to 6 and higher scores indicate greater disability.
  (2) Secondary outcomes:
  i. Symptomatic intracranial hemorrhage risk (defined by European Collaborative Acute Stroke Study II criteria^[23]^);
  ii. Incidence of emerging major vascular events within 6 months. Major vascular AEs include ischemic stroke, hemorrhagic stroke, transient ischemic episodes, etc;
  iii. All-cause mortality.

### 2.9 Safety reporting

AEs were defined as any adverse or unexpected signs, symptoms, or disease occurred during the study period with or not associated with experimental treatment between the 2 groups. The investigator should take appropriate measures to ensure patient safety and track all AEs results until the condition returned to normal or stability.

SAEs are events that may cause permanent or serious disability, or even life-threatening. If a SAEs occurs, the lead investigator shall be notified by telephone at the first time (within 24 hours). The lead investigator then reported the SAEs to the Safety Monitoring Board (an independent committee of trial experts that will focus on safety monitoring to ensure the study meets the highest ethical standards and ensure patient safety) and contacted relevant experts for consultation to develop treatment options.

During the study period, all reported or observed AEs or SAEs would be recorded in the CRF, including the nature of each event, time of onset, duration, intensity, evaluation of its cause, treatment protocol, and outcomes.

### 2.10 Data management and quality control

Any modifications or changes to the protocol would be re-approved through the formal procedures of the Ethics Committee of our hospital. The Safety Monitoring Committee would regularly review research progress and ensure that the research complies with standard procedures. Trained researchers would collect study data and record them in the CRF. To ensure the reliability of the data, all patients’ personal information would be collected and stored in a separate storage room limited to researchers from the research team and must be accessed by 2 or more members to protect pre-, on-, and posttrial confidentiality. All information from the patient would not be disclosed and transmitted without the patient's written permission.

### 2.11 Statistical analysis

The efficacy assessment would be determined by the full analysis set and the per-protocol set, and the security assessment would be based on the security set. Statistical evaluation of full analysis set would follow the principle of intent-to-treat. Estimation of missing values for the main variables was performed using the last observation carried forward method. The collected data were statistically analyzed using the SPSS 22.0 software. Chi-square test was used for counting data. Mean ± standard deviation ($\bar{\text{x}}$ ± S) was used for measurement data, independent sample *t* test was used for normal distribution, and Mann–Whitney *U* test was used for skewness distribution. The difference was considered statistically significant when *P* < .05.

## 3 Discussion

AIS is a disease that is characterized by high recurrence rate, high fatality rate, and high disability rate^[8]^ due to various reasons (cerebral atherosclerosis, increased blood viscosity, foreign body, vascular congenital malformation, vascular endothelial thickening, arterial plaque formation and rupture, inflammation and adipokines, arterial stenosis, even occlusion, thrombosis), resulting in sharply reduced distal cerebral artery blood perfusion, insufficient cerebral blood supply and oxygen supply, and even brain tissue necrosis,^[24]^ resulting in neurological impairment and cognitive dysfunction.^[25,26]^ The treatment of AIS should not only realize early vascular recanalization and restore brain tissue irrigation, but also pay attention to the recovery of nerve function and the possibility of vascular events recurrence.

Danhong injection is extracted from Danshen (*Radix Salviae Miltiorrhiae*) and Honghua (*Flos Carthami*), and it is a certified Chinese patent medicine. Although previous studies have demonstrated that Danhong injection reduces neurological impairment caused by acute stroke, improves blood microcirculation, improves clinical efficiency, but also significantly reduces the disability rate and mortality associated with cerebral infarction.^[15,27,28]^ However, these studies lack long-term follow-up, and AIS neurological function recovery is a slow process, the main recovery period within 2 to 6 months after the onset, and no clinical studies to evaluate the impact of Danhong injection on neurological recovery during the recovery period and the incidence of adverse vascular events during this period. Moreover, these studies also have methodological defects, such as insufficient randomization, no double-blind and placebo control, and insufficient sample size, and these factors can bias the results, which also reduces the reliability of clinical evidence. The design of reasonable randomized controlled trials is the gold standard for evaluating the clinical efficacy and safety of TCM.^[29]^ Therefore, this study is proposed to explore the effect of Danhong injection on neurological recovery and AEs in AIS patients using a standard randomized controlled experiment and long-term follow-up.

There are also some deficiencies in this study: first of all, although our follow-up time is 6 months, which is much longer than other clinical studies on Danhong injection, it is still unable to objectively evaluate the impact of Danhong injection on cardiovascular AEs and survival cycle of AIS patients. Therefore, in real studies we may extend the follow-up time; secondly, this study belongs to a single-center study, patients included in the study may be regionalized; finally, since this study was to observe the efficacy of Danhong injection on the basis of standard therapy, therefore, we were still unable to determine whether Danhong injection could be used as a complete alternative treatment for AIS.

## Author contributions

**Conceptualization:** Junfeng Gao and Xiangzhong Shao.

**Data curation:** Junfeng Gao and YiXiang Guan.

**Formal analysis:** Xiangzhong Shao and YiXiang Guan.

**Funding acquisition:** Junfeng Gao.

**Software:** YiXiang Guan and Juxiang Mei.

**Supervision:** Xiangzhong Shao and Juxiang Mei.

**Writing – original draft:** Junfeng Gao and YiXiang Guan.

**Writing – review & editing:** Xiangzhong Shao and Juxiang Mei.
